# A Longitudinal Study on Feeding Behaviour and Activity Patterns of Released Chimpanzees in Conkouati-Douli National Park, Republic of Congo

**DOI:** 10.3390/ani3020532

**Published:** 2013-06-07

**Authors:** Amandine Renaud, Aliette Jamart, Benoit Goossens, Caroline Ross

**Affiliations:** 1 HELP Congo (Habitat Ecologique et Liberté des Primates), BP 335, Pointe Noire, Congo; E-Mail: aliettejamart@yahoo.fr; 2 Centre for Research in Evolutionary Anthropology, Department of Life Sciences, Roehampton University, Holybourne Avenue, London SW15 4JD, UK; E-Mail: c.ross@roehampton.ac.uk; 3 Danau Girang Field Centre, c/o Sabah Wildlife Department, Wisma Muis, 88100 Kota Kinabalu, Sabah, Malaysia; 4 Organisms and Environment Division, Cardiff School of Biosciences, Cardiff University, Biomedical Sciences Building, Museum Avenue, Cardiff CF10 3AX, UK; E-Mail: goossensbr@cardiff.ac.uk

**Keywords:** chimpanzees, reintroduction, release, conservation, activity budget, diet, Republic of Congo

## Abstract

**Simple Summary:**

Wild chimpanzee populations are dramatically declining due to anthropogenic pressure. One way of increasing wild population numbers and/or repopulating areas where local extinction has occurred is to release captive animals. HELP Congo was the first project to successfully release wild-born orphan chimpanzees in their natural environment. We studied the behaviour of eight released chimpanzees over eight years. Over time, they modified their behaviour, suggesting long-term behavioural and ecological adaptations. This suggests that successful release programmes may reinforce existing populations of endangered species.

**Abstract:**

Wild chimpanzee populations are still declining due to logging, disease transmission and hunting. The bushmeat trade frequently leads to an increase in the number of orphaned primates. HELP Congo was the first project to successfully release wild-born orphan chimpanzees into an existing chimpanzee habitat. A collection of post monitoring data over 16 years now offers the unique opportunity to investigate possible behavioural adaptations in these chimpanzees. We investigated the feeding and activity patterns in eight individuals via focal observation techniques from 1997–1999 and 2001–2005. Our results revealed a decline in the number of fruit and insect species in the diet of released chimpanzees over the years, whereas within the same period of time, the number of consumed seed species increased. Furthermore, we found a decline in time spent travelling, but an increase in time spent on social activities, such as grooming, as individuals matured. In conclusion, the observed changes in feeding and activity patterns seem to reflect important long-term behavioural and ecological adaptations in wild-born orphan released chimpanzees, demonstrating that the release of chimpanzees can be successful, even if it takes time for full adaptation.

## 1. Introduction

The great apes have experienced a significant population reduction in the past 20 to 30 years and they continue to be threatened by poaching and habitat destruction [1,2]. Great apes are also frequently found in the pet trade, leading to large numbers of orphan animals arriving in sanctuaries. Attempts have been made to release wild-born orphan primates back into their natural environment [3,4,5,6]. Although long-term data on release programs are rare [5,6], such studies are vital if we are to assess the value of releases for either conservation or animal welfare [7].

Reintroduction has been defined as “an attempt to establish a species in an area which was once part of its historic range, but from which it has been extirpated or become extinct” [2]. Reintroducing wild animals in natural environments can be a useful tool for conservation because it can restore biodiversity [8], provide an important demographic and genetic reservoir for endangered species, and decrease the threat of extinction in endangered species [9]. In addition, releasing animals into areas where there is a wild population may also be done for a variety of other reasons, including animal welfare and supplementing wild populations where numbers are low; such cases may be referred to as reinforcement, supplementation or restocking [2]. Below we refer to both reintroduction and reinforcement programmes as release programmes.

Since the 1960s, several attempts have been made to release great apes into the wild [3,4,5,6,10,11,12,13,14,15,16,17,18,19]. Only a few projects have resulted in continued survival and successful reproduction of released individuals; in the 1960s in Rubondo forest (Tanzania—here chimpanzees were released onto islands, not in forested areas), and in the 1990s onwards in Conkouati-Douli National Park (Republic of Congo), and, more recently, in 2008, in Haut Niger National Park (HNNP) in Guinea (both latter projects released chimpanzees into forested areas) [3,6,12,13]. However, post monitoring of released primates is poorly documented in the literature [4,12,14,15,16,17,18] and it is not always possible to determine the reasons why success in release varies. 

One of the released projects that has had positive results and has been described as a successful release was initiated in 1996 by HELP Congo, in the Conkouati-Douli National Park, Republic of Congo [14]. A 2005 study found that 37 released chimpanzees had an average survival rate of 62%, with several released females reproducing, mating with wild as well as with released males [14]. A study of the same population showed that, following release, the chimpanzees adjusted their social structure to become stabilized into a single community with a fission–fusion system [18]. Between 1996 and 2012, a total of 53 chimpanzees were released (17 are still monitored, 15 are dead, one has been returned to one of the sanctuary islands, 20 are no longer monitored but are thought to have integrated into wild communities). Of the 17 monitored individuals, five females had two infants aged between two and 10 years. 

Monitoring of these animals has resulted in several studies of their ecology. Farmer *et al.* (2006) studied the behaviour in the 14 months post-release of 16 chimpanzees released between 1996 and 1999 [12] and found that the released chimpanzees had activity budgets within the range of wild chimpanzees, although grooming time was low compared with wild populations in similar habitats. The same animals ate more than 239 plant parts post-release, including at least 122 species, of which 62 were identified [12]. 

Here we extend the analyses of feeding ecology of these animals using post-release monitoring data from 1997–1999 and 2001–2005 and investigate possible adaptations in feeding ecology and activity patterns in released chimpanzees over a post-release time period of up to eight years. In particular, we address the following questions: (1) Are there changes in the feeding ecology in released chimpanzees over the investigated time period? (2) Are there changes in activity patterns in released chimpanzees over the investigated time period? 

## 2. Methods and Study Subjects

### 2.1. Study Site

**Figure 1:**
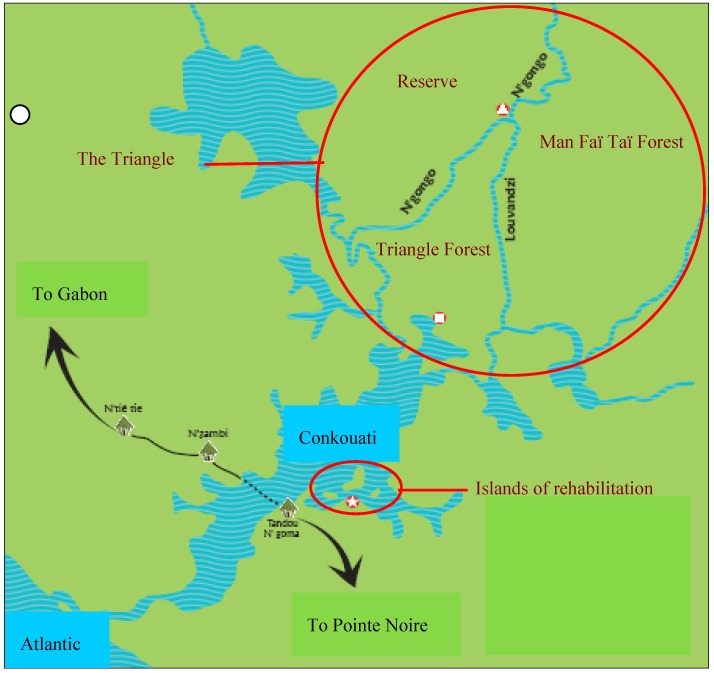
Map of the release site Triangle within the sanctuary of HELP Congo (the release site so called the “Triangle” of 22 km² area, is composed of three sites, the Triangle forest, the Man Faï Taï forest and the Reserve. Chimpanzees are free ranging, using natural bridges to cross the rivers).

The study site, the “Triangle”, is located in the North of the Conkouati lagoon in the Conkouati-Douli National Park (3°33'–4°02'S; 11°10'–11°40'E), Republic of Congo (Figure 1). The “Triangle” has an area of 22 km², and was chosen in 1996 by HELP Congo to be the release site of wild-born orphan chimpanzees due to its abundance of food, especially fruits, its low population density of wild chimpanzees (0.17–0.33 individuals/km^2^), and absence of people [12,14,20]. The rainy season is concentrated from October to May and there is a dry season from June to September [21].

Six hundred species of plants have been identified, including around 100 species known to be eaten by wild chimpanzees [20]. The “Triangle” is surrounded by two rivers, but is not fully isolated as natural bridges occur, which allow the released chimpanzees to travel freely and to have access to two other areas of the Conkouati-Douli National Park: the Man Fai Tai forest and the Reserve (Figure 1).

### 2.2. Study Animals, Data Collection and Analyses

We investigated the feeding ecology and activity patterns in eight wild-born orphaned chimpanzees. All were separated from their mothers, who were presumably killed for the bush meat market, and arrived as infants in captivity, aged from approximately six months to three years old (Table 1). They were rescued by the Congolese organization HELP Congo and were transferred to three small islands, of 5–10 km², in the middle of the lagoon of Conkouati (Figure 1) in 1991. They did not have contact with wild chimpanzees on these islands. The chimpanzees were released between 1996 and 1999 at the “Triangle” (details given in [12,14]) (Table 1). Chimpanzees selected for the release were between 6 and 11 years (age at release: 6.3–10.9 years, mean 8.3 years, s.d. 1.6 years). Young animals without dependant infants were chosen as it was thought that they would have more chance to integrate with wild communities at this stage of sexual maturity [14] To minimise possible risk of disease transmission, the released animals chosen had all been living on the isolated islands since 1991 and were healthy; health checks were also carried out prior to release [14,20,22]. Table 1 gives information on the characteristics of released studied chimpanzees. 

**Table 1:** Characteristics of released focal chimpanzees.

ID	Sex	Arrival age (years)/ Estimated date of birth/Date arrived sanctuary)	Release age (years)/ Date release	Number data blocks ^(a)^ (dates data collection)	Date death (cause)	Time since release to 2009 or death (years)	Offspring born in Triangle ^(b)^	No. infants known up to 2009
Bounie	F	5.0 Jan 1987–Jan 1992	9.8 Nov 1996	4 (1997–1998)	–	13	Yes	1
Choupette	F	1.8 Nov 1989–Aug 1991	6.9 Nov 1996	10 (1997–1999, 2003–2004)	–	13	Yes	3
Jeanette	F	3.2 Jun 1988–Aug 1991	8.3 Nov 1996	10 (1997–1999, 2003–2004)	–	13	Yes	1
Mekoutou	M	2.0 Jun 1990–Jun 1992	6.3 Nov 1996	11 (1997–1999, 2003–2005)	Apr 2006 (unknown)	10	Yes	1
Rosette	F	1.5 Feb 1990–Aug 1991	6.9 Feb 1997	6 (1997–1999)	–	12	–	–
Koutou	M	2.2 Jun 1989–Aug 1991	9.6 Feb 1999	12 (1999, 2001–2005)	Mar 2009 (Wild attack)	10	–	–
Chinois	M	2.2 Jun 1989–Aug 1991	11.0 Jun 2000	10 (2001–2005)	–	9	–	–
Bilinga	M	0.4 Jun 1993–Nov 1993	8.0 Jun 2001	4 (2002–2004)	Oct 2004 (*Oesopha-gostomum* infection)	3	–	–

^(a)^ Data were means for 3-month blocks (see text for details); ^(b)^ Genetic tests were confirmed for males’ offspring [23].

A protocol of post release monitoring was established to gather information on the process of release [12]. All released chimpanzees were fitted with radio-collars (Telonics Inc., USA). Following their release, the animals were followed from nest to nest, seven days a week, by a trained team of several Congolese field assistants and international volunteers. Data collected included diet, activity budget, nesting, ranging behaviour, female reproductive status, health status and interactions with wild conspecifics [14]. Whenever possible, observers maintained a minimum distance of 7 m between themselves and the chimpanzees to reduce disease transmission [2]. Gradually, as the chimpanzees grew independent, the staff increased the security distance between themselves and the chimpanzees [22] to 7 meters minimum.

Data analysed here were collected on feeding ecology and activity patterns from 1997–1999 and 2001–2005. Observers recorded behaviour using focal sampling carried out with instantaneous observations every 10 minutes [24], recording information onto check sheets using standard activity and dietary definitions (Table 2). For feeding behaviour, plant taxa and food parts eaten were also noted (Table 2). Observations were made mostly from dawn to dusk (06:00 to 18:00), but sometimes for shorter periods, depending on weather conditions and the available number of field assistants. Once integrated into wild communities, females were not the subject of focal follows and were only observed interacting with reintroduced males. 

For the statistical analyses of the long-term activity and feeding pattern in the released chimpanzees, only data fulfilling the following criteria were included: (1) a minimum of continual data collection for 10 days/month for at least 24 months post-release (range 25–67 months), and (2) overall data availability over a period spanning at least three years post-release (range 3–13 years). Using these criteria we extracted data from the long-term records for four females and four males aged from 6 to 11 years at release (Table 1). Data for each animal were analysed for 10 days per month for two three-month periods in each year (March–May and September–November) (Table 1). As data collection quality and quantity varied, these months were chosen to maximize good quality data, rather than ensure equal coverage of wet and dry seasons. Data were available for 61 full three month blocks, and an additional 6 blocks, where data for only two of the three months were available, were also included. 

**Table 2:** Activity and dietary definitions.

Activity budget	Definition
Travel	Moving (walking, running, jumping) on the ground or in the trees, climbing or descending
Allogrooming	Grooming another chimpanzee
Rest	Lying, sleeping, sitting, not moving
Feed	Removing food items from a substrate, eating, chewing, swallowing
Other	All the activities that do not correspond to those listed above, e.g., aggression, play, copulation, veterinary interventions, displays, hunting, nest building and coprophagy
**Dietary items**	**Items included**
Plant	Fruit (Fr.), leaf (L.), flower (Fl.), seed (Se.), and stem (St.)
Insect	Ants, larvae, caterpillars, termites and grasshoppers
Meat	Birds, pangolins, and squirrels
Other food items	Sap (exudates eaten from tree trunks/branches), bark, soil, clay and honey

Plant species and plant parts eaten were recorded on the observation sheets. Each of these parts are analysed as separate food items in the study. When a plant taxon was not known it was listed as Unknown. A food list was made from this information (Table 3), including the vernacular name (when possible) and the scientific name of the plants eaten by the chimpanzees [25]. Plants eaten were classified to genus level in most cases and to species level where possible (Table 3). 

The means for 10 days of each month per animal were calculated to give monthly percentages for each of the behaviours. These were then averaged again for each three-month period. This gave 67 period means for the eight animals (range 4–12 per animal). This method thus allows changes of behaviour to be tracked over time (*i.e.*, at six-monthly intervals). 

The ages and time since release of each animal were recorded for the mid-month for each three-month period, *i.e.*, in April and October.

**Table 3:** Plant items eaten, listed in order of importance in diet: Fruit (Fr.), Leaf (L.), Flower (Fl.), Seed (Se.), Stem (St.). Herb: herbaceous plant up to 5 m height [25,26,27]. Shrub: 1–2 m height; 2 m height & 10 cm in diameter; 10–20 cm in diameter [25]. Tree: 20–50 cm in diameter, 50–100 cm in diameter, more than 100 cm in diameter [25]. Liana: supple stems with contortion, tendrils, hooks of great heights. Woody liana & herbaceous liana differ with the woodiness of the stem [25].

Genus/Species	Family	% diet	Local name	Part consumed					Plant type
				Fl.	Fr.	L.	Se.	St.	
*Irvingia gabonensis*	Irvingiaceae	15			x		x		Tree
*Vitex*	Verbenaceae	14		x	x	x		x	Tree
*Staudtia*	Myristicaceae	10	Niove		x		x		Tree
*Nauclea*	Rubiaceae	9	Bilinga		x				Tree
*Elaeis guineensis*	Palmae	8			x	x			Tree
*Millettia*	Papilionaceae	6		x		x			Tree
*Aframomum*	Zingiberaceae	6	Ntoundou	x	x		x	x	Herb
*Dialium*	Caesalpiniaceae	5	Dakar		x	x			Tree
*Grewia*	Tiliaceae	5			x		x		Tree
*Landolphia*	Apocynaceae	5	Malombo		x		x		Liana
*Scytopetalum*	Scytopetalaceae	4			x				Shrub
*Santiria*	Burseraceae	3			x				Tree
*Hexalobus*	Annonaceae	2	Vadou		x				Tree
*Ipomoea*	Convolvulaceae	2			x	x			Herb
*Warneckea*	Melastomataceae	2			x		x	x	Herb
*Diospyros*	Ebenaceae	1			x	x	x		Tree
*Ficus*	Moraceae	1			x	x			Shrub
*Saccoglottis gabonensis*	Humiriaceae	1	Ozouga		x				Tree
*Urera*	Urticaceae	1				x			Woody liana
*Afzelia*	Caesalpiniaceae	<1				x			Tree
*Agelaea*	Connaraceae	<1		x		x			Woody liana
*Alchornea*	Euphorbiaceae	<1	Mukukusa		x		x		Shrub
*Allanblackia*	Clusiaceae	<1			x				Tree
*Ancistrophyllum*	Palmae	<1	Mukawa					x	Liana
*Annona*	Annonaceae	<1			x				Shrub
*Asplenium*	Aspleniaceae	<1				x			Herb
*Berlinia*	Caesalpiniaceae	<1	Ebiara	x		x			Tree
*Bridelia*	Euphorbiaceae	<1			x				Tree
*Canthium*	Rubiaceae	<1			x				Shrub
*Chytranthus*	Sapindaceae	<1				x			Tree
*Cissus*	Vitaceae	<1				x			Liana
*Citrus*	Rutaceae	<1			x				Tree
*Cleistopholis patens*	Annonaceae	<1			x				Tree
*Cola*	Sterculiaceae	<1			x	x	x		Tree
*Costus*	Zingiberaceae	<1	Mussangavulu		x	x		x	Herb
*Dacryodes*	Burseraceae	<1	Safou		x				Tree
*Desplatsia*	Tiliaceae	<1		x	x				Tree
*Diogoa*	Olacaceae	<1			x				Tree
*Eremospatha*	Palmae	<1	Mbamba		x			x	Liana
*Enantia*	Annonaceae	<1			x				Tree
*Gnetum africanum*	Gnetaceae	<1	Mfumbu		x				Tree
*Guibourtia*	Caesalpiniaceae	<1			x		x		Tree
*Haumania*	Marantaceae	<1			x				Herb
*Hibiscus*	Malvaceae	<1				x			Herb
*Hypselodelphis*	Marantaceae	<1	Mangongolo		x				Herb
*Klainedoxa*	Irvingiaceae	<1			x				Tree
*Macaranga*	Euphorbiaceae	<1	Msamsa		x				Tree
*Manihot*	Euphorbiaceae	<1	Manioc		x				Tree
*Mapania*	Cyperaceae	<1			x			x	Herb
*Marantochloa*	Marantaceae	<1				x			Herb
*Milicia excelsa*	Moraceae	<1	Iroko		x				Tree
*Mimosa*	Mimosaceae	<1			x	x			Liana
*Musanga*	Moraceae	<1	Musenga		x				Tree
*Myrianthus*	Moraceae	<1			x				Tree
*Palisota*	Commelinaceae	<1			x			x	Herb
*Parkia*	Mimosaceae	<1		x	x	x			Tree
*Pentaclethra*	Mimosaceae	<1	Tibossi		x	x			Tree
*Pentadesma butyracea*	Clusiaceae	<1			x				Tree
*Polyalthia*	Annonaceae	<1			x				Tree
*Pseudospondias*	Anacardiaceae	<1	Ngarila		x	x			Tree
*Salacia*	Celastraceae	<1			x	x			Tree
*Strychnos*	Loganiaceae	<1			x				Woody liana
*Symphonia globulifera*	Clusiaceae	<1				x			Tree
*Trachyphrynium*	Marantaceae	<1						x	Herb
*Trichoscypha*	Anacardiaceae	<1	Mvuta		x				Tree
*Uapaca*	Euphorbiaceae	<1			x				Tree
*Uvaria*	Annonaceae	<1			x				Tree
*Uvariastrum pierreanum*	Annonaceae	<1			x	x			Tree
*Xylopia*	Annonaceae	<1	Moukana		x				Tree
*Treculia*	Moraceae	<1			x				Tree
*Pycnanthus*	Myristicaceae	<1	Mulomba		x				Tree
*Ongokea gore*	Olacaceae	<1			x				Tree
*Podococcus*	Palmae	<1			x				Tree
*Raphia*	Palmae	<1	Mahouhou		x	x		x	Tree
*Panda oleosa*	Pandaceae	<1				x			Tree
*Leptoderris*	Papilionaceae	<1			x	x			Shrub
*Pterocarpus*	Papilionaceae	<1			x				Tree
*Passiflora*	Passifloraceae	<1			x				Herb
*Oxytenanthera abyssinica*	Poaceae	<1	Bambou			x			Herb
*Porterandia*	Rubiaceae	<1			x				Tree
*Sherbournia*	Rubiaceae	<1			x				Tree
*Pancovia*	Sapindaceae	<1			x				Tree
*Baillonella*	Sapotaceae	<1	Moabi		x				Tree
*Sterculia*	Sterculiaceae	<1	Ikirulanga	x	x	x	x		Tree
*Glyphaea brevis*	Tiliaceae	<1				x			Tree

### 2.3. Statistics

Age data were normally distributed but time since release were not (Kolmogorov-Smirnov test p < 0.05). Most activity budget and diet data were normally distributed, except for percentage of flowers, seed, meat and insects which are non-parametric (Kolmogorov-Smirnov test p < 0.05), Correlation tests were conducted to investigate relationships between behaviour, diet (consumption of each food type) and time since release (months) and age (months) of the subjects (Pearson’s Correlation Coefficient for parametric data and Spearman’s Correlation Coefficient for non-parametric data). 

## 3. Results and Discussion

### 3.1. Results

#### 3.1.1. Feeding Patterns

We identified a total of 86 plant taxa (Table 3) from at least 42 plant families that were eaten by the eight chimpanzees. The chimpanzees were observed to feed on all plant parts, the preferred plant parts being fruits (58%), followed by leaves (18%), stem (16%) and seeds (3%). Insects, soil, and meat only represented a small part (less than 3% in total) of the diet.

**Figure 2:**
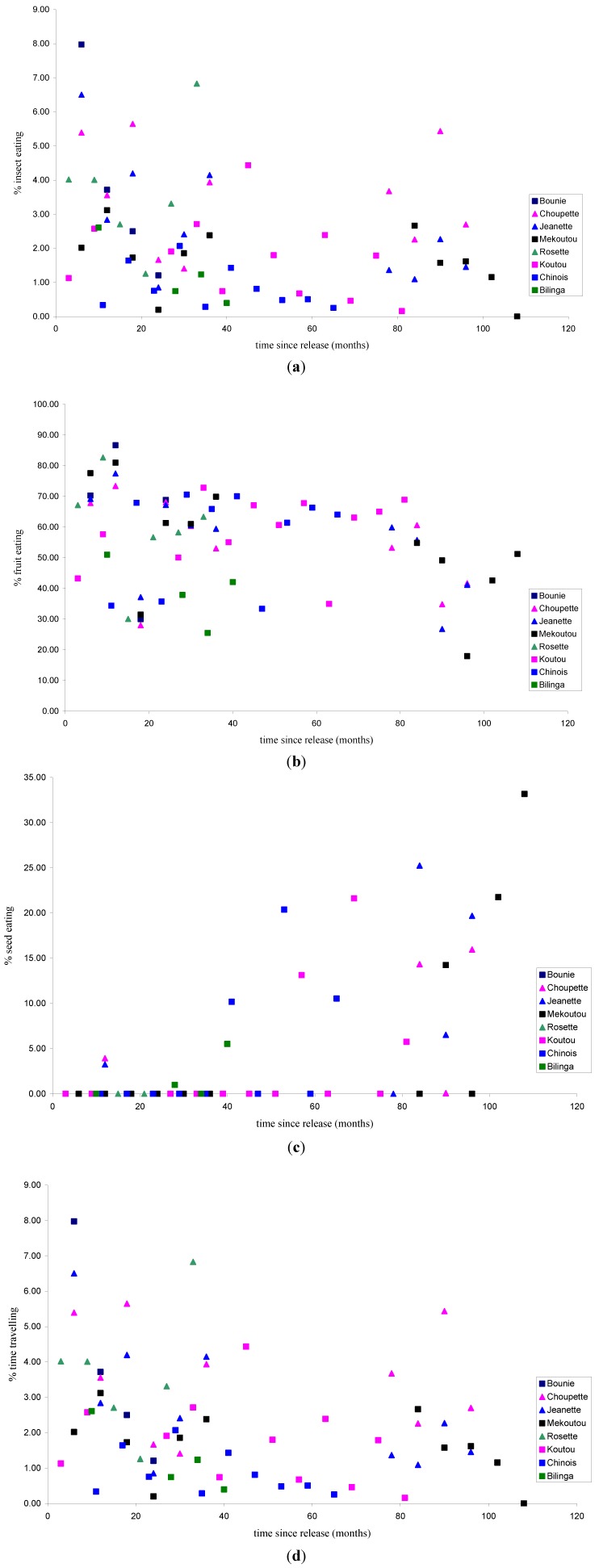
Significant relationships with time since release and (**a**) % insect eating; (**b**)% fruit eating; (**c**) % seed eating; (**d**) % time travelling. Each individual is represented as a separate symbol. Females are shown as triangles and males as squares.

**Figure 3:**
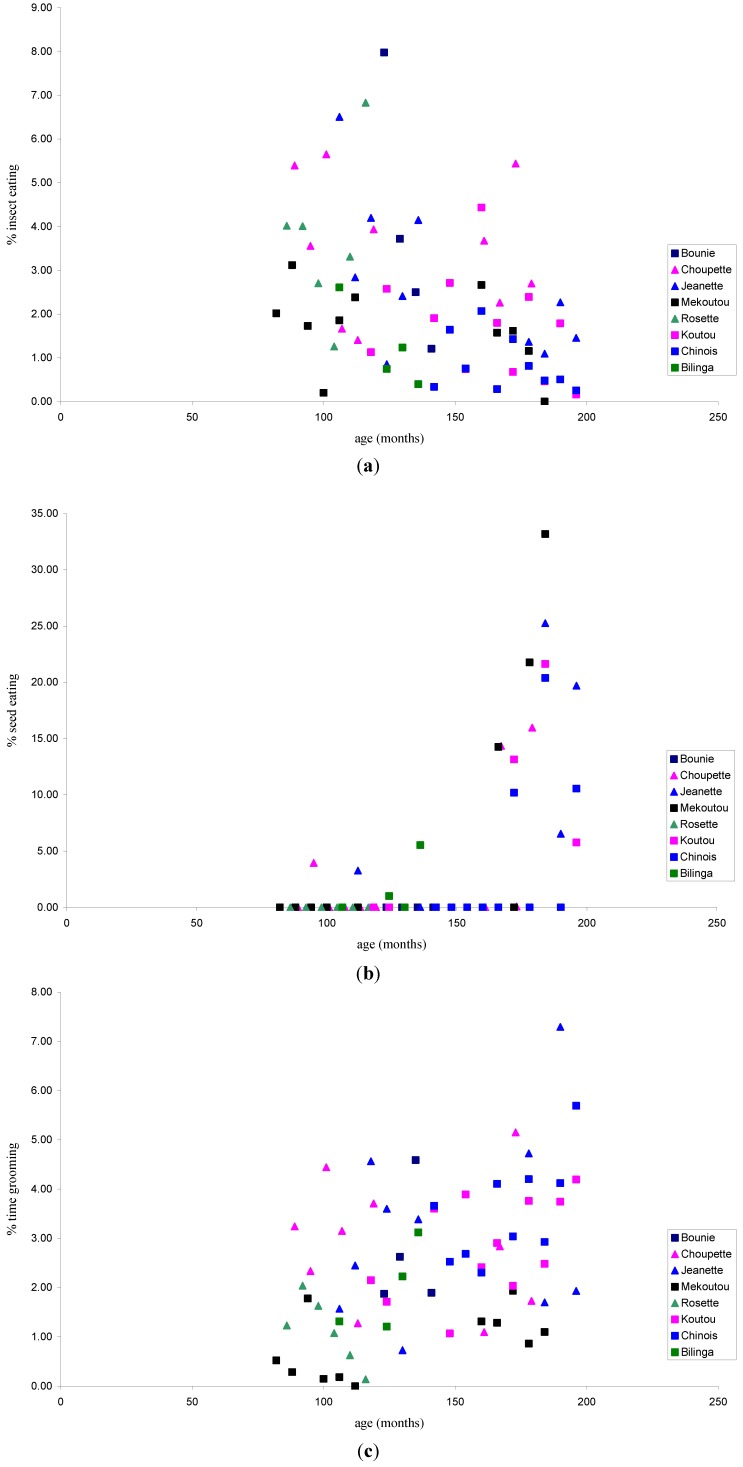
Significant relationships with age and (**a**) % insect eating; (**b**) % seed eating; (**c**) % time grooming. Each individual is represented as a separate symbol. Females are shown as triangles and males as squares.

When comparing the feeding ecology in the released chimpanzees over time, we found a negative correlation between time since release and fruit consumption (Figure 2, r_s_ = −0.311, p = 0.010, n = 67) but not between fruit consumption and age (r = −0.198, p = 0.108, n = 67). Time since release and insect consumption were also negatively correlated (Figure 2, r_s_ = −0.350, p < 0.004, n = 67) as was age and insect consumption (Figure 3, r_s_ = 0.429, p < 0.001, n = 67). Conversely we found a positive correlation between seed consumption and time since release (Figure 2, r_s_ = 0.567, p < 0.001, n = 67) and with seed consumption and age (Figure 3, r_s_ = 0.544, p < 0.001, n = 67). There were no correlations with leaf consumption and either age (r = −0.139, p = 0.261) or time since release (r_s_ = −0.165, p = 0.182, n = 67), nor with flower consumption and age (r = 0.080, p = 0.519, n = 67) ortime since release (r_s_ = 0.039, p = 0.752, n = 67), nor with meat consumption and age (r = −0.017, p = 0.890, n = 67) or time since release (r_s_ = 0.040, p = 0.749, n = 67).

When each of the feeding measures is considered for individual animals the same trends were seen but none of the correlations were statistically significant, although the directionalities of some effects were significant. One-tailed T-tests were used to investigate whether correlation coefficients for individuals were significantly different from zero; age and insect eating have a negative correlation that is significantly different from zero (p = 0.024, n = 7), age and seed a positive correlation (p > 0.001, n = 7), and time since release and fruit consumption a trend towards a negative correlation (p = 0.09, n = 7). It should be noted that correlation coefficients for either age or time since release and any behaviour will be exactly the same *within* individuals as the age and time since release are completely correlated within individuals. 

#### 3.1.2. Activity Patterns

The chimpanzees spent most of their time feeding (51.51%), followed by resting (26.46%), travelling (14.90%), and grooming (2.49%). Over time, we found a marginally insignificant negative correlation between the time since release and time spent on travelling (Figure 2, r_s_ = −0.235, p = 0.056, n = 67), which became significant when one extreme outlier was removed (r_s_ = −0.292, p = 0.017, n = 66). There was no significant correlation between age and time spent travelling (r = −0.081, p = 0.514, n = 67). There was a positive correlation between the age of animals and time spent on grooming (Figure 3, r = 0.439, p < 0.001, n = 67) but the similar positive relationship was not significant for time since release and time spent on grooming (r_s_ = 0.161, p = 0.193, n = 67). No significant correlations were found between feeding and age (r = −0.045, p = 0.720, n = 67) or time since release (r_s_ = −0.004, p = 0.972, n = 67), nor with resting and age (r = −0.023, p = 0.851, n = 67) or time since release (r_s_ = 0.163, p = 0.187, n = 67).

When each of the behaviours is considered for individual animals, the same trends were seen but none of the correlations were statistically significant, although the directionalities of some effects were significant. One-tailed T-tests were used to investigate whether correlation coefficients for individuals were significantly different from zero; age and travel has a negative correlation that is significantly different from zero (p = 0.050, n = 7), although age and grooming shows a trend towards a positive correlation this is not significant (p = 0.194, n = 7).

### 3.2. Discussion

Our study reveals interesting changes in feeding ecology and activity patterns in released chimpanzees over the investigated time period of eight years. We observed similarities with the food items consumed by wild chimpanzees. The high levels of fruit in the diet (58%) followed by leaf (18%) and then stem (16%) is similar to that found in wild studies where fruit is also the main dietary item [16,28,29] and to the diet of the island released population at Ipassa in Gabon [15].

The percentage of the main food types, fruit and leaf, in the diet of the released chimpanzees is within the range found in wild chimpanzees. Fruit consumption of the released animals (58%) is within the range of wild chimpanzee e.g., at Lopé, Gabon (66%), Assirik, Senegal (58%), Bossou, Guinea (52%), Gombe, Tanzania (43%), Mahale, Tanzania (32%) and Semliki, Uganda (39%) [30,31,32,33] and the released chimpanzees of Ipassa, Gabon (67%) [15]. Leaf consumption (18%) is also within the wild chimpanzee range, e.g., Assirik (10%), Lopé (12%), Bossou (18%), (27%), Semliki (30%), Mahale (37%) and similar to the released at Ipassa (10%) [15,30,31,32,33]. 

Wild populations of chimpanzees are reported to consume variable numbers of plants, from only 45 plant species being recorded in Semliki, Uganda [33] to 198 plant species recorded in Mahale, Tanzania [32] and more than 200 plant species recorded in Bossou population (Guinea) [28]. Thus, it appears that the diversity of diet in the released chimpanzees of Conkouati-Douli (86 plant taxa, 42 plant families) is within the range found in wild animals. 

Previous studies at Conkouati-Douli showed that the released chimpanzees added several feeding plants to their diet, presumably by a trial and error learning, during the first years of their release [20]. Farmer (2006) reported that, three years after their release, the chimpanzees fed on different parts of more than 122 plant species, with the main diet being made up of 44% fruits and 26% leaves. Over eight years, our study showed fruit and leaves are similarly important but overall, fruits make up the greater part of the diet, and leaves a lesser part. These feeding differences might be explained by the time scale over which data were collected or by variation in annual food availability [34,35,36]. In 2006, Farmer *et al.* [12] collected data equally over both wet and dry seasons but we have more data on the wet months; this may partly explain the higher fruit and lower leaf consumption in our study, as the rainy season (October to May) corresponds to fruit abundance and the dry season (June to September) corresponds to fruit scarcity [22]. Unfortunately, we do not have data on food availability over time, so we cannot be sure whether inter-annual variation in food availability is also important.

Our study does show that, when the same months are compared across years, fruit consumption decreased over time. One reason for the change in diet over time may be that during the captive period and early pre-release state (spent in semi-liberty on islands), the animals were mainly being fed with fruits (e.g., banana, papaya, figs) and therefore may have preferentially chosen to feed on fruit soon after release [12,20]. The decline in fruit eating is most probably because, in later years, the dietary repertoire expanded to include a greater range of food types, in particular seeds, as is found in wild chimpanzees [33,34,35,36,37].

The decline in insect consumption over time is surprising as it might be assumed that finding and capturing insects would become easier, and thus, performed more frequently, for the animals as they matured and spent more time in the wild. However, the increase in seed eating (which may be explained by the chimpanzees learning to find and process such foods) may partly explain this as both seeds and insects have a high protein content; thus an increase in seed eating may be balanced by less insect feeding. An alternative explanation is that the chimpanzees did not decrease the total amount of insects they ate but instead managed to eat them more efficiently and thus, spent less time processing these foods (this may be particularly true for feeding on social insects using tools). Investigating this would require collection of detailed data on foraging efficiency.

Surprisingly, there is a smaller number of consumed fruit taxa in our study as compared with an earlier study [12]. The lower number of taxa may be partly due to our study not including all months of the year, and to differences in the accuracy of data recording. However, the large difference (86 *vs.* 122 fruits species), and the overall decline in time spent feeding on fruits over the years, suggests that the difference may be a real one. This might be explained by the animals first sampling a wide range of fruits but then excluding some as they became more adept at finding preferred foods. Optimal foraging theory predicts that animals should forage in such a way as to maximize their net energy intake per unit time [38,39]. That means the chimpanzees should behave in such a way as to search and find, handle and consume fruits with the most net energy input (energy content/digestive turnover) while spending the least amount of energy and time in doing so. This could be an interesting line of new research. 

Another explanation for the decline in number of fruit taxa consumed might be the learned avoidance of specific fruits due to a high content of secondary plant chemicals. It is known that primates sometimes avoid fruits with high contents of secondary plant chemicals such as alkaloids and tannins and the chimpanzees may have learnt this over time [40]. Although chimpanzees may be able to tolerate higher tannin levels by eating soil as a detoxification strategy [41], using such behaviour to allow feeding on less palatable foods may need to be learnt. We have no evidence for this hypothesis at present and testing it would require further research, both on feeding behaviour and secondary compounds in dietary items. 

The observed changes in activity patterns, with a decreased time spent on travelling and an increase in time spent on social activities (grooming) may also indicate adaptations to the new environment. We suggest that after the first years of being released, the chimpanzees had to travel more intensively due to the necessity of exploring their new habitat, finding new food patches, and defining their home range within an area inhabited by wild chimpanzees. Once the home range was defined and food patches identified, travelling time for food searching and feeding time decreased. While travelling decreased, the amount of time spent on allogrooming increased as the animals matured, which is of high importance for social stabilization [18].

## 4. Conclusions

In conclusion, the changes in activity pattern as well as the changes in feeding ecology in the released chimpanzees seem to reflect important long-term behavioural and ecological adaptations to their environment. It is widely accepted that primates can adjust their behaviour to different ecological and dietary niches [36]. Here we demonstrate that orphan chimpanzees, which were separated from their natal group at an early age, lacking maternal contact and natal social culture, have succeeded in adapting to a new environment over time. This study suggests that reintroduction and/or reinforcement of existing populations may be possible and have benefits both to animal welfare and to conservation efforts.

Further studies should focus on the nutritive components of the items eaten (and not eaten) by the released chimpanzees as well as the learning processes in the food choice of the new released chimpanzees, as this information was not included in the data sheet of the studied subjects. Furthermore, it would be interesting to study the energy expenditure and feeding efficiency in released chimpanzees to investigate changes towards a more optimized energy balance over years.
